# Preclinical Evaluation of the Tumorigenic and Immunomodulatory Properties of Human Bone Marrow Mesenchymal Stromal Cell Populations with Clonal Trisomy 5

**DOI:** 10.1155/2022/1613636

**Published:** 2022-08-19

**Authors:** Maria Susana Joya Marodin, Juliana A. Godoy, Raquel M. Alves-Paiva, Kelen Alvarez, Thiago Giove Mitsugi, Ana Cristina Victorino Krepischi, Nelson Hamerschlak, Maria Augusta Tezelli Bortolini, Rodrigo Castro, Andrea T. Kondo, Jose Mauro Kutner, Oswaldo Keith Okamoto

**Affiliations:** ^1^Human Genome and Stem Cell Research Center, Department of Genetics and Evolutionary Biology, Institute of Biosciences, University of Sao Paulo, Sao Paulo, Brazil; ^2^Department of Hemotherapy and Cellular Therapy, Albert Einstein Israelite Hospital, Sao Paulo, Brazil; ^3^Department of Gynecology, Federal University of Sao Paulo, Sao Paulo, Brazil

## Abstract

Cytogenetic aberrations may emerge in human mesenchymal stromal cells (MSC) during ex vivo expansion for cell therapy. We have detected clonal trisomy 5 in two distinct autologous MSC products expanded from bone marrow which, based on the current quality control criteria, could not be released for clinical use. Although a safety concern, it is still unclear to what extent recurrent aneuploidies detected in MSC products may affect the threshold for neoplastic transformation or the medicinal properties of these cells. We have carried out an exploratory preclinical study to evaluate these MSC products with clonal trisomy 5, regarding their oncogenic and immunomodulatory potential. Cell population growth *in vitro* was reduced in MSC cultures with clonal trisomy 5 compared with the population growth of their euploid MSC counterparts, based on a lower cumulative population doubling level, reduced cell proliferation index, and increased senescence-associated beta-galactosidase activity. Subcutaneous injection of clinically relevant amount of MSC population, either with or without clonal trisomy 5, did not generate tumors in immunodeficient mice within a follow-up period of six months. Most importantly, MSC population with clonal trisomy 5 kept immunomodulatory properties upon interferon gamma (IFN*γ*) licensing, displaying overexpression of *IDO*, *CXCL9*, *CXCL10*, and *CXCL11*, in a similar fashion than that of IFN*γ*-licensed euploid MSC. Our findings suggest that bone marrow MSC products with clonal trisomy 5 may retain their therapeutic potential, based on poor tumor initiating capability and preserved immunomodulatory potency. This preclinical evidence may further support the definition of release criteria of autologous MSC products for cell therapy under critical clinical scenarios. This trial is registered with Clinical Study registration number: RBR-29x2pr.

## 1. Introduction

Mesenchymal stromal cells (MSC) have long been applied in cell therapy trials for the treatment of several diseases. Although MSC can be harvested from many vascularized tissues, their low abundance requires ex vivo expansion to achieve suitable doses for clinical use [1]. However, culture conditions during expansion can favor the emergence and/or selection of cells with chromosomal aberrations [2]. Indeed, several studies have reported detection of chromosomal alterations in ex vivo expanded MSC [3–7]. For this reason, cytogenetic analysis is required as part of the release testing for clinical grade MSC.

Nonetheless, there is a need for standard harmonized criteria for releasing MSC products for infusion into patients. Experts of the Cell Products Working Party and the Committee for Advanced Therapies of the European Medicines Agency have recommended performing karyotype of 20 metaphases and an exclusion limit of two metaphases (10%) with clonal chromosomal aberration [8]. Despite these safety recommendations, it is still unclear to what extent certain defined genetic aberrations detected in MSC may affect their medicinal properties and threshold for transformation.

The neoplastic transformation is a multiple step process involving the accumulation of many genetic alterations. The presence of a chromosomal alteration can be a signal of genomic instability, an important hallmark of cancer. Genomic instability is the source of new genetic alterations which lead to the acquisition of new hallmarks of cancer [9]. However, not all chromosomal aberrations will trigger cell transformation. Abnormal cells can also undergo apoptosis or become senescent, avoiding the expansion of genetically unstable clones [10].

There are two reports of spontaneous transformation of human MSC during *in vitro* culture. These transformed cells were also able to give rise to tumors when inoculated into mice [11, 12]. However, these transformed MSCs already showed abnormal behavior *in vitro* likely due to the multiple chromosomal alterations detected, including translocations, deletions, and duplications involving several chromosomes. Usually, cells presenting such abnormal behavior during manufacturing would be promptly discarded by good manufacturing practice (GMP) quality control.

In rare cases, MSC exhibiting normal culture behavior and potency after final expansion may have few clonal genetic aberrations detected by release testing. In this practical scenario, it is uncertain whether these MSC products with clonal aberrations would be prone to transformation *in vivo* and, therefore, unsafe for clinical use. Proper tumorigenicity assessment of aneuploid MSC requires chronic follow up studies. This knowledge is fundamental to determine acceptable safety criteria for releasing cell therapy products.

Trisomy 5 is a recurrent aneuploidy found in ex vivo expanded MSC [5–7]. Recently, we detected trisomy 5 in two independent autologous MSC products expanded from bone marrow samples of patients enrolled in a clinical trial for treatment of urinary incontinency. Due to current cytogenetic criteria, these MSC products could not be released for clinical use. Here, we report an ensuing exploratory preclinical evaluation of these MSC products with clonal trisomy 5 regarding their oncogenic and immunomodulatory potential.

## 2. Methodology

### 2.1. Samples and Cell Culture

All MSC were obtained from bone marrow of patients after informed consent. Cells were processed either at the Cell Therapy Laboratory from the Albert Einstein Hospital (São Paulo, Brazil) or at the Human Genome & Stem Cell Research Center from the University of São Paulo (São Paulo, Brazil). Two MSC samples with trisomy 5 (sample ID: T5-50, T5-53), manufactured under GMP conditions, and two MSC samples with normal karyotype (sample ID: M10, M12) were included in the preclinical evaluation. Samples T5-50 and T5-53 were obtained from 50 and 53-year-old patients, respectively, enrolled in a clinical trial for treatment of urinary incontinency. M10 and M12 were obtained from donors under 55 years of age. The study was approved by the institutional Ethics Committee (CAAE: 3496419.6.0000.5464). Cells were cultured in DMEM Low Glucose (Gibco) supplemented with 10% of Fetal Bovine Serum (FBS) and 1% of Anti-Anti (100X) Antibiotic-Antimycotic solution (Gibco) at 37°C. The culture medium was changed twice a week. Cells were dissociated at 80% confluence with TrypLE™ Express (Gibco) and seeded again at low density (6.000 cells per cm^2^) in a new cell culture flask. MSC identity was confirmed by flow cytometric immunophenotyping, in agreement with the International Society for Cell and Gene Therapy's recommendation [13] (Supplementary figures S1-S4, supplementary table S1).

### 2.2. Cytogenetic Analysis

G-banding karyotype of MSC analyzed 20 metaphases per sample. MSCs with two or more (≥10%) metaphases with clonal chromosome aberration were considered with abnormal karyotype. Briefly, cells were cultured in a T-75 flask until reaching 70-80% cell confluence and then synchronized at G1 cell cycle phase by a 20 h incubation in culture medium without FBS. After 30 hours in medium supplemented with FBS, cells were incubated for 4 hours with KaryoMAX™ Colcemid™ (Gibco) (0.05 *μ*g/mL), dissociated with TrypLE, lysed with hypotonic solution (0.075 M KCL) prewarmed for 30 minutes at 37°C, and fixed in Methanol: Acetic acid (3 : 1) solution. Metaphase slides were made by dropping cells on glass slides followed by G-banding staining. Chromosome aberrations were described according to the International System for Human Cytogenomic Nomenclature [14].

A chromosomal microarray analysis (CMA) based on array-CGH was performed for evaluation of copy number variation (CNV). Genomic DNA samples were extracted from cell cultures using QIAamp DNA Mini Kit, according to the manufacturer's protocol. Array-CGH was performed with a 180 K oligonucleotide platform (Agilent Technologies, California, USA) according to the manufacturer's protocol, using a control DNA obtained from a male. Analyses were carried out using the software Nexus Copy Number (Biodiscovery) with the FASST2 Segmentation algorithm, based on at least three consecutive aberrant probes, threshold log_2_ test Cy3/control Cy5 ratio of |0.1| for gains and losses (allowing the detection of mosaicism), and |1.2| for amplifications and homozygous losses. Common germline CNVs were excluded based on the Database of Genomic Variants (http://dgv.tcag.ca/dgv/app/home). The human genome annotation was the GRCh37/hg19 from the Genome Browser at the University of California Santa Cruz (UCSC) (https://genome.ucsc.edu/). The cytogenetic analyzes were performed at passages 4 to 6, as part of the release tests, and repeated at later passages in which cells were harvested for use in the preclinical *in vivo* experiment assessing tumorigenicity.

### 2.3. Cell Population Growth

MSC growing pattern during expansion was evaluated by cumulative Population Doubling Level (PDL). Cells were first kept in culture medium without FBS for 20 hours to allow cell cycle synchronization and then cultured with complete culture medium supplemented with 10% FBS. When reaching 80% confluence, cells were dissociated with trypsin and seeded at low density (6.000 cells/cm^2^) into a new flask. PDL was estimated by the following formula: PDL = log10(Cf/Ci)∗3.32, where Ci is the cell number that were plated at the beginning of incubation time and Cf is the cell number at the end of the incubation time. Doubling Time (DT) was calculated by the following formula: DT = *T*∗ln2/ln(Cf/Ci), where *T* is the incubation time in days. Formulas are according to ATCC [15].

### 2.4. Cell Proliferation

Cell proliferation rate was determined with the Click-iT™ EdU Cell Proliferation Kit for Imaging Alexa Fluor (Invitrogen). Cells were plated in triplicate in 24-well plates; after 48 hours, cells were incubated with EdU for 4 hours. The following steps were in accordance with the manufacturer's protocol. Images were captured using the EVOS M5000 Imaging System microscope. Cells and proliferative nuclei were counted with the ImageJ software.

### 2.5. Cellular Senescence

Cell senescence assays were based on the senescence-associated beta-galactosidase (SA-*β*-Gal) activity. Both the number of positive cells for the enzyme activity and the levels of enzyme activity were measured. Cells were seeded in triplicate in 24-well plates. After 48 hours, a colorimetric assay was carried out using the Senescence *β*-Galactosidase Staining kit (Cell Signaling), following the manufacturer's protocol. Images were captured with EVOS XL Core Imaging System microscope. Positive cells for SA-*β*-Gal were counted, and the percentage of senescent cells was calculated. A fluorescence assay (CellEvent™ Senescence Green Detection Kit, Invitrogen) was also performed to determine the intensity of cellular SA-*β*-Gal activity. Enzymatic activity was determined 48 hours after plating cells in chamber slides. Images were captured with the Zeiss Axio Imager Z1 ApoTome fluorescence microscope, and fluorescence intensity was determined with ImageJ. The cell fluorescence was calculated as follows: FC = total fluorescence–(cell area∗background fluorescence).

### 2.6. Tumorigenicity Assay

To verify whether MSC with trisomy 5 can undergo spontaneous transformation *in vivo* and give rise to tumors, 13-week-old female Balb c/NUDE mice were subjected to subcutaneous injection of 1 × 10^5^ cells into the right flank. The experimental groups (*n* = 3/group) in this assay were defined by the type of cell injected (M10, M12, T5-50, or T5-53). As a positive control for tumor development, a group of mice received injection of 1 × 10^5^ USP7-AT/RT cells (atypical teratoid rhabdoid tumor cell line) which display tumor-initiating capability [16]. Animals were followed for 185 days, weighed weekly, and tumors measured with a digital caliper. Tumor volume (mm^3^) was calculated with the following formula: (larger diameter × smaller diameter^2^)/2. All efforts were made to minimize animal suffering as proposed by the Guide for the Care and Use of Laboratory Animals [17]. This preclinical study was approved by the Institutional Animal Experimentation Ethics Committee of Bioscience Institute from University of São Paulo (CEUA 358/2019).

### 2.7. Potency Analysis

To evaluate MSC immunomodulatory properties, MSC cells were licensed with interferon gamma (IFN*γ*) and immunomodulatory response was indicated by relative expression of *IDO*, *CXCL9*, *CXCL10*, and *CXCL11* [18]. Cells were incubated in culture medium with 40 ng/mL of INF*γ* for 48 hours. After this period, cells were lysed, and total RNA was extracted with Rneasy Micro Kit (Qiagen). cDNA was synthesized using SuperScript™ III Reverse Transcriptase (Invitrogen), and real-time PCR was performed using SYBR Green (Power SYBR Green PCR Master Mix, Applied Biosystems) and specific primers for *IDO*, *CXCL9*, *CXCL10*, *CXCL11*, and *GAPDH* (primer sequences provided in Supplementary table S2). Expression data was normalized by *GAPDH*, and M10 was the reference sample.

### 2.8. Statistical Analysis

Analysis of variance of parametric data was performed with One-Way ANOVA and Tukey's post hoc test. Analysis of nonparametric data was performed with Kruskal-Wallis and Dunn's multiple comparisons test. All analyses were performed in GraphPad Prism 8 software. *P* values < 0.05 were considered statically significant (^∗^*P* < 0.05;  ^∗∗^*P* < 0.01;  ^∗∗∗^*P* < 0.001;  ^∗∗∗∗^*P* < 0.0001).

## 3. Results

### 3.1. Cytogenetic Characteristics of MSC Populations

**Figure 1:**
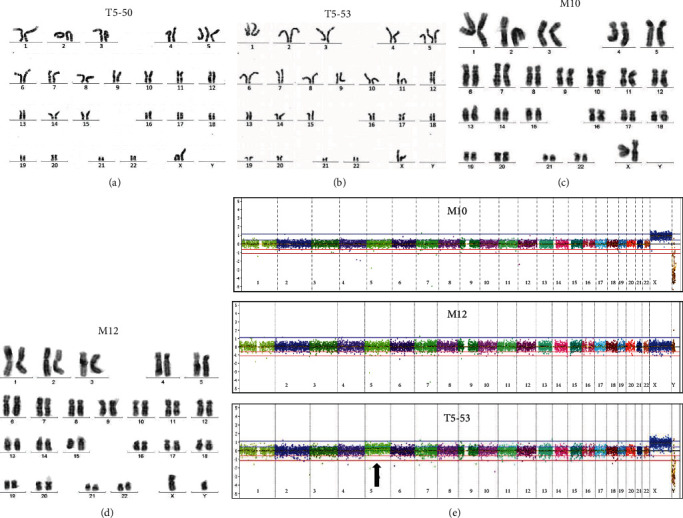
Cytogenetic characterization of distinct human MSC populations after ex vivo expansion. (a–d) Karyotyping results of the four investigated MSC cell lines. (a) MSC sample T5-50 karyotype 47,XX,+5. (b) MSC sample T5-53 karyotype 47,XX,+5. (c) MSC sample M10 karyotype 46,XX. (d) MSC sample M12 karyotype 46,XY. (e) CNV profiles of three MSC cell lines showing the results observed by CMA. Microarray probes are plotted along the *X*-axis according to their genomic coordinates and each color represents a chromosome (indicated below), from 1 to 22, *X* and *Y* (from the short to the long arm). The *Y*-axis represents the log^2^ scale of the copy number ratio MSC cell lines/control (values close to 0 indicate regions with similar copy number between tested and control samples; positive values represent gains; negative values represent losses). The arrow shows the detected trisomy of chromosome 5 in mosaic.

G-banding karyotype analyses reveled that MSC samples T5-50 and T5-53 had 10% and 30% of metaphases with trisomy 5, respectively (Figures 1(a) and 1(b)). MSC sample M10 exhibited 75% of normal metaphase (46, XX) and 25% (5/20) of nonclonal chromosome losses. MSC sample M12 showed 95% (19/20) of normal metaphases (46, XY) and one abnormal metaphase (5%) (Table 1) (Figures 1(c) and 1(d)). Even though some chromosomal anomalies were detected in M10 and M12 samples, they presented the quality parameters required by the current recommendation for cell therapy product release due to the lack clonal chromosomal abnormalities [8]. Random loss of chromosomes usually occurs as a technical artifact after the metaphase spreading in slide preparation [6]. CMA did not detect rare CNVs in the MSC samples M10 and M12 (Figure 1(e)). Conversely, the array-CGH data of the MSC sample T5-53 revealed the presence of mosaicism for chromosome 5 trisomy. This result, taken together with the karyotype obtained for the T5-53 sample in previous passages, confirmed the trisomy 5 in mosaic (about 10% of the cell population at passage 9). CMA was not performed for T5-50 sample because there were not enough viable cells for DNA extraction to yield sufficient genomic material for the molecular analysis.

### 3.2. MSC Cultures with Clonal Trisomy 5 Were Mostly Comprised of Low Proliferative and Senescent Cells

**Figure 2:**
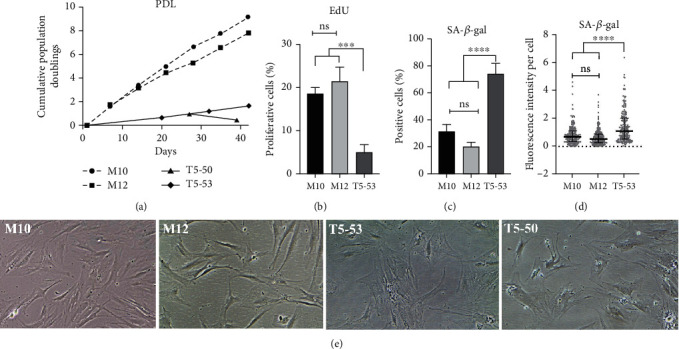
Characterization of human MSC cell cultures. (a) Cumulative population doublings during cellular expansion ex vivo. Passage interval of samples: M10: P5 to P10; M12: P7 to P12; T5-50: P6 to P8; T5-53: P7 to P10. (b) Percentage of proliferative cells. One-Way ANOVA and Tukey's multiple comparisons test. (c) Percentage of cells with SA-*β*-galactosidase activity; 100 cells analyzed per well. One-Way ANOVA and Tukey's multiple comparisons test. (d) Intensity of SA-*β*-galactosidase activity per cell; cell fluorescence intensity (CF) calculated with ImageJ by CF = total fluorescence–(cell area × background intensity). Kruskal-Wallis and Dunn's multiple comparisons test. (e) Comparison of cellular morphology of M10 (Passage 8) and M12 (Passage 10) with T5-50 (Passage 7) and T5-53 (Passage 8) samples. T5-50 and T5-53 cells with typical flattened and enlarged senescent morphology. All pictures were captured using 100x magnification. Assays were performed in triplicate, at the same passage. Error bars represent standard deviation. ^∗∗∗^*P* value < 0.001; ^∗∗∗∗^*P* value < 0.0001; ns: statistically nonsignificant. M10 and M12: euploid human bone marrow MSC samples; T5-50 and T5-53: human bone marrow MSC samples with clonal trisomy 5.

The analysis of MSC population growth *in vitro* showed a lower cumulative PDL for cultures of cells harboring clonal trisomy 5, compared with the PDL of euploid MSC. After a period of 35 days of expansion, MSC samples T5-50 and T5-53 showed, respectively, 0.4 and 1.7 population doublings, while samples M10 and M12 had 7.7 and 6.5 doublings, respectively (Figure 2(a)). Moreover, sample T5-50 underwent a population reduction after 27 days of the beginning of the assay, lacking expansion potential thereafter. This abrupt decrease in T5-50 cell population restricted their use in the downstream cellular and biochemical assays. In contrast, MSC samples M10 and M12 displayed a steady PDL throughout the expansion period. Of note, all MSC samples from patients enrolled in the clinical trial (including the ones harboring trisomy 5) attained sufficient amount of cells for the established dose at passage interval P4-P6. The significant impairment of cell expansion of MSC with trisomy 5 was assessed at later culture passages. The PDL analysis was performed at passage intervals P5 to P10 for sample M10, P7 to P12 for sample M12, P6 to P8 for sample T5-50, and P7 to P10 for sample T5-53.

Such differences in MSC population growth *in vitro* were partly due to differences in cell proliferation rates and cellular senescence. Proliferation of MSC T5-53 cells was significantly lower than those of euploid MSC cells (*p* < 0.001), a behavior that is not expected in cells with oncogenic potential. The percentage of proliferating cells in the MSC T5-53 culture was 5.4 ± 1.6, compared with 18.4 ± 1.6 and 21.3 ± 3.4 percent of proliferating cells in MSC M10 and M12 cultures, respectively (Figure 2(b)).

In addition, detection of SA-*β*-galactosidase activity indicated that MSC T5-53 culture was mostly comprised by senescent cells. Based on the colorimetric enzyme assay, the number of senescent cells in MSC T5-53 culture was 2.4-fold and 3.7-fold higher than the amount in MSC M10 and M12 cultures, respectively (*p* < 0.0001). The percentage of positive cells for SA-*β*-galactosidase activity was 74 ± 7.8 in T5-53 culture, 31 ± 5 in M10 culture, and 20 ± 2.9 in M12 culture (Figure 2(c)). In agreement with the colorimetric data, the fluorescence-based enzyme assay also showed that the level of cellular SA-*β*-galactosidase activity was significantly higher in MSC T5-53 culture than in MSC M10 and M12 cultures (*p* < 0.0001) (Figure 2(d)). Despite the lack of sufficient material to assay MSC T5-50 culture, these cells displayed typical senescent morphology with enlarged and flattened shape, as opposed to the typical fibroblast-like elongated morphology of healthy MSC found in M10 and M12 cultures (Figure 2(e)). Cell proliferation and senescence assays were performed at passages P9 and P10 for samples M10 and M12, respectively, and at passages P8 and P9 for samples T5-50 and T5-53, respectively.

### 3.3. Presence of Clonal Trisomy 5 in MSC Cultures Did Not Increase Risk of Tumorigenicity

**Figure 3:**
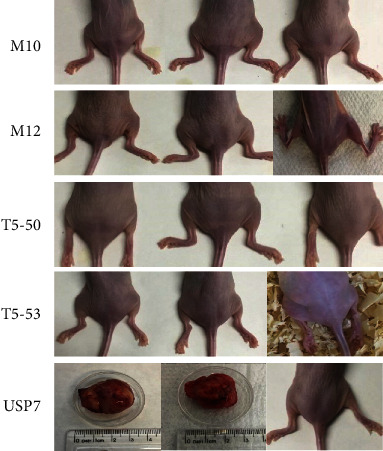
Tumorigenic potential of ex vivo expanded MSC. Images of tumors or flank region of animals at the time of euthanasia. M10 and M12: euploid human bone marrow MSC samples; T5-50 and T5-53: human bone marrow MSC samples with clonal trisomy 5; USP7: tumorigenic human ATRT cell line.

Subcutaneous injection of MSC in the flank of nude mice did not result in the formation of tumors. This result was verified either with injections of MSC harboring trisomy 5 (MSC samples T5-50 or T5-53) or with injections of euploid cells (MSC samples M10 or M12). Animals were monitored for 185 days regarding presence of tumor in the injection site, body weight, and overall behavior. As a technical control for *in vivo* tumorigenesis, a group of animals received subcutaneous injections of human tumor cells (USP7 cells). Two out of three animals in this latter group developed tumors that became visible after 38 and 45 days of cell inoculation (Figure 3). The presence of tumors affected locomotion and behavior of the animals, which also consistently lost body weight and had to be euthanized at days 87 and 94 postinjection.

In addition, two animals that received MSC had to be euthanized before the end of the long follow-up period of 185 days postinjection. One animal of the group injected with MSC M12 cells was euthanized 171 days after cell inoculation due to consistent weight loss. Also, in the group injected with MSC T5-53 cells, one animal was euthanized 143 days after cell inoculation due to loss of the movement of hind legs (Table 2).

### 3.4. MSC Cultures with Clonal Trisomy 5 Displayed Preserved Immunomodulatory Potential

**Figure 4:**
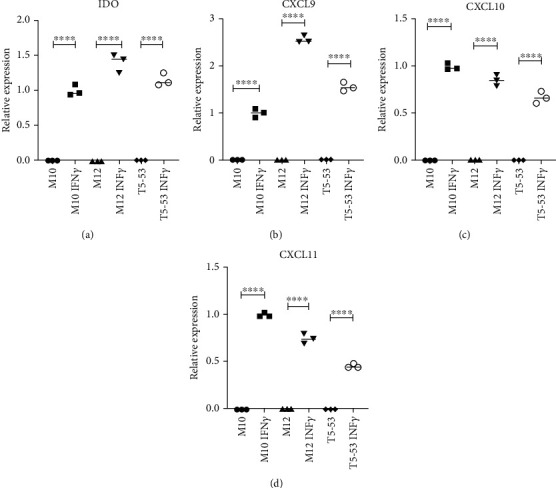
Relative expression of cytokines related to MSC immunomodulatory response, before and after stimulation by IFN-*γ*. (a) *IDO* mRNA relative expression. (b) *CXCL9* mRNA relative expression. (c) *CXCL10* mRNA relative expression. (d) *CXCL11* mRNA relative expression. Values were normalized by GAPDH expression, using M10 IFN*γ* licensed as the reference sample. ^∗∗∗∗^*P* < 0.0001. M10 and M12: euploid human bone marrow MSC samples; T5-50 and T5-53: human bone marrow MSC samples with clonal trisomy 5.

Immune potency of MSC samples was evaluated by their ability to be activated by the proinflammatory cytokine IFN-*γ*. Surprisingly, upon IFN-*γ* stimulation, a similar activation response was detected in MSC culture with clonal trisomy 5, compared to cultures of euploid MSC. For all MSC culture samples, the expressions of *IDO*, *CXCL9, CXCL10*, and *CXCL11* in IFN-*γ*-licensed cells were significantly higher than the respective expression in cells without stimulation (*p* < 0.0001). This data suggests retention of immunomodulatory properties and a high proportion of senescent cells in MSC culture harboring clonal trisomy 5 (Figure 4).

## 4. Discussion

Studies that have followed MSC *in vivo* found out that most cells are undetectable after 30 days of their inoculation [7, 12] and that they have low engraftment potential [19]. It is also well known that lack of attachment to the extracellular matrix induces cell cycle arrest in normal cells [20]. Thus, the short residence time and low engraftment of exogenous MSC after systemic inoculation *in vivo* decrease the likelihood of their tumor formation. Nonetheless, few MSCs are able to fix into tissues [19] and one cannot exclude the possibility of transformation of these engrafted MSCs, especially if they carry chromosomal alterations.

In our work, MSC with trisomy 5 displayed lower proliferative potential and reduced population growth *in vitro* than euploid MSC. This result differs from that of Tarte and colleagues [7], who reported similar population doubling rates in cultures of MSC with trisomy 5 and cultures of MSC with normal karyotype. Notably, trisomy 5 disappeared fast and spontaneously in MSC cultures of the latter study, probably due to negative selection of aberrant cell clones during cell culture passages [6, 7]. This phenomenon may possibly explain the different MSC growth pattern observed in our study, which detected MSC clones with trisomy 5 along all period of ex vivo expansion.

In agreement with this scenario, our data indicates that MSC with trisomy 5 became senescent earlier than euploid MSC, based on detection of hallmarks of cellular senescence, including increased SA-*β*-Gal activity, loss of proliferative potential, morphological alterations, and a senescence-associated secretory phenotype (SASP) [21]. The hypothesis that trisomy 5 may contribute to MSC senescence is supported by the observation that aneuploid cells usually adopt a default behavior characterized by slow proliferation, proteotoxic stress, genetic instability, and acceleration of cell aging [10, 22]. Aneuploidy can also induce a DNA damage response, activating p53 that triggers senescence or apoptosis [22, 23].

However, some aneuploidies may overcome these senescent-related mechanisms and confer selective growing advantage to adult stem cells, such as trisomy 8 [3] and trisomy 12 [24]. Such specific aneuploidy effects on cell behavior are determined by the combination of active genes present in the altered chromosome. There are few data about specific responses related to trisomy 5. It is believed to be a deleterious chromosome alteration, since there is no record of babies born alive with trisomy 5 and only seven records are available for mosaic trisomy 5 individuals [25]. Among these mosaic cases, there are patients with multiple congenital anomalies, intrauterine growth restriction and growth delay. In addition, the 5q35 region harbor genes known to be dosage sensitivity, and the 5q35 duplication is associated with a phenotype named reverse Sotos Syndrome, characterized by low stature, microcephaly, and growth retardation [26]. Indeed, trisomy and tetrasomy 5 cells show a slow population growing [27]. Also, senescent cells have the potential to stimulate the senescence of neighboring healthy cells by secretion of soluble factors such as TGF-*β* [28]. These evidences point to an inhibitory effect of clonal trisomy 5 on the proliferative cell rate in MSC cultures.

Although chromosomal alterations are present in 90% of solid tumors, trisomy 5 is not common in tumors, including sarcomas [24], the type of tumor generated by cells of mesenchymal origin [11, 12]. When trisomy 5 is present in tumor cells, it is usually associated with many other chromosomal alterations, likely as a result of genetic instability intrinsic to neoplasms [29]. In this sense, trisomy 5 does not seem to be a key cytogenetic alteration contributing to tumor initiation and progression. In our *in vivo* preclinical study, human bone marrow-derived MSC with trisomy 5 did not show enhanced tumorigenic potential when compared to their euploid counterparts. Given that tumor progression takes time because it is a multiple step process, our study followed up mice injected with a clinically relevant amount of MSC for six months. Considering age correlation between mice and humans, this follow-up period in mice is equivalent to 14 years of follow up in humans [30]. Even after this long period of observation in our study, no tumors were detected in animals injected with MSC, either euploid or carrying trisomy 5. Most studies that have evaluated the oncogenic potential of MSC had a short follow-up, usually only one or two months, which may not be sufficient to detect potential long term oncogenic effects [7, 11, 12]. In the clinical setting, MSC with trisomy 5 [7] or trisomy 7 [31] has already been injected into humans that were followed up for two and five years, respectively, with no signs of tumor formation. In a recent study, MSCs harboring trisomy 5 were reported incapable of generating colonies in soft agar, therefore lacking the ability of transformed cells to grow in an anchorage independent manner [32]. Thus far, to the best of our knowledge, there are no evidences that nontransformed human MSC with trisomy 5 in mosaic give rise to tumors after *in vivo* injection.

Interestingly, despite the association with senescent cell phenotype, presence of clonal trisomy 5 did not impair the immunomodulatory properties of MSC cultures. Upon IFN*γ* licensing, MSC cultures with clonal trisomy 5 showed an immunomodulatory transcriptional profile [18], similar to that of IFN*γ*-licensed euploid MSC, characterized by an overexpression of *IDO*, *CXCL9*, *CXCL10*, and *CXCL11*. This result is in agreement with other reports of immune functionality in replicative senescent MSC [33] and indicates preservation of therapeutic potential involving the immunomodulatory mechanism of action. Since our assay detected variation in transcript levels, it would be important to confirm variation of the corresponding enzyme and secreted proteins.

## 5. Conclusions

Altogether, our preclinical findings reveal that the presence of clonal trisomy 5 in bone marrow MSC cultures does not seem to be associated with increased tumorigenic potential nor with lack of immunomodulatory properties. This knowledge may support medical decision regarding exceptional release of autologous MSC products for cell therapy under critical clinical scenarios, when new harvesting of biological material and/or new MSC manufacturing is not feasible. It is important, however, that the results from this study be not extrapolated to other types of chromosome alterations. Additional similar studies are necessary to better understand the potential clinical consequences of other common chromosomal alterations found in MSC products.

## Figures and Tables

**Table 1:** Cytogenetic characterization by karyotype and CMA of human bone marrow MSC samples included in the study.

MSC sample	Karyotype (number of metaphases)	Passage at karyotyping	CMA	Passage at CMA
M10	46,XX[15]/44,XX,-8,17[1]/ 45,XX,-5[1]/44,XX,-5,-18[1]/ 45,XX,-22[1]/45,XX,-21[1]	9	arr(1-22,X)x2	9
M12	46,XY[19]/44,X,-10[1]	10	arr(1-22)x2, (XY)x1	10
T5-50	46,XX[18]/47,XX,+5[2]	4	NA	---
T5-53	46,XX[14]/47,XX,+5[6]	6	arr(X)×2,(5)×3 [0.1]	9

Nomenclature according to International System for Human Cytogenomic Nomenclature (ISCN, 2016). NA: not available (due to low number of viable cells after cell culture).

**Table 2:** Tumor incidence, overall survival, and adverse signs in nude mice after 185 days of subcutaneous injection of MSC or tumor cells. Animals Survival after cell inoculation (p =0.2247).

Cell sample injected	Cell description	Frequency of tumor-bearing animals/total animals evaluated	Survival at day 185 post-injection	Adverse clinical signs
T5-50	Human MSC cells harboring trisomy 5	0/3	100%	None
T5-53	Human MSC cells harboring trisomy 5	0/3	67%	1 animal with reduced mobility
M10	Human euploid MSC	0/3	100%	None
M12	Human euploid MSC	0/3	67%	1 animal with consistent weight loss
USP7	Human ATRT tumor cells	2/3	33%	2 tumor-bearing animals with reduced activity and excessive weight loss

## Data Availability

All data supporting this study are included within the article. Raw data are available upon request to the corresponding author.

## Supplementary Materials

Supplementary Materials Figures S1-S4 contain data on MSC immunophenotyping. Table S1 presents the percentage of cells expressing typical MSC surface markers in samples M10, M12, T5-50, and T5-53. Table S2 contains the primer sequences used in the gene expression assay.
