# Supplementary material for: Preclinical Evaluation of the Tumorigenic and Immunomodulatory Properties of Human Bone Marrow Mesenchymal Stromal Cell Populations with Clonal Trisomy 5
Source: Stem Cells Int. 2022 Aug 19;2022:1613636. doi: 10.1155/2022/1613636 (PMC9417782; doi:10.1155/2022/1613636)
Supplement: Supplementary Materials — Figures S1-S4 contain data on MSC immunophenotyping. Table S1 presents the percentage of cells expressing typical MSC surface markers in samples M10, M12, T5-50, and T5-53. Table S2 contains the primer sequences used in the gene expression assay. [file 1613636.f1.docx]

**Supplementary material**

**MSC characterization**

All MSC samples showed a typical MSC immunophenotype, in accordance with ISCT requirements^14^. MSC samples has high expression of CD105, CD90, CD73, CD44 and CD166 and low expression of CD45, HLA-DR and CD31 (Supplementary figure S1- S4) (Supplementary table S1).

Supplementary figure S1- Results from MSC sample T5-50 flow cytometry. Control cells without antibody in gray and antibody marked cells in red.

Supplementary figure S2- Results from MSC sample T5-53 flow cytometry. Control cells without antibody in gray and antibody marked cells in red.

Supplementary figure S3- Results from MSC sample M10 flow cytometry. Control cells without antibody in blue and antibody marked cells in pink.

Supplementary figure S4 - Results from MSC sample M12 flow cytometry. Control cells without antibody in blue and antibody marked cells in pink.

*Supplementary Table S1 – Percentage of flow cytometry detection of surface markers.*

| Target | M10 (%) | M12 (%) | T5-50 (%) | T5-53 (%) |
| --- | --- | --- | --- | --- |
| CD105 | 99.0 | 99.0 | 65.3 | 91.2 |
| CD73 | 99.9 | 99.8 | 91.9 | 95.9 |
| CD90 | 99.9 | 99.8 | 95.9 | 99.6 |
| CD44 | 99.6 | 99.7 | 99.2 | 96.5 |
| CD166 | 99.7 | 99.3 | 93.4 | 99.5 |
| CD31 | 0.8 | 0.2 | 4.2 | 0.4 |
| CD34 | 32.8 | 10.7 | 0.7 | 2.4 |
| CD45 | 0.3 | 0.5 | 4.6 | 0.1 |
| HLA-DR | 1.4 | 1.48 | 5.3 | 7.4 |

Supplementary Table S2 – Primers sequence for rt-PCR.

| **Target** | **Orientation** | | **Sequence** |
| --- | --- | --- | --- |
| *IDO1* | | *Forward* | 5’-AGATGTCCGTAAGGTCTTGCC-3’ |
|  |  | *Reverse* | 5’-ACTGCAGTCTCCATCACGAAA-3’ |
| *CXCL9* | | *Forward* | 5’-TTCTGATTGGAGTGCAAGGAAC-3’ |
|  |  | *Reverse* | 5’-CCTTCACATCTGCTGAATCTGGG-3’ |
| *CXCL10* | | *Forward* | 5’-CCACGTGTTGAGATCATTGCT-3’ |
|  |  | *Reverse* | 5’-TGCATCGATTTTGCTCCCCT-3’ |
| *CXCL11* | | *Forward* | 5’-GCTACAGTTGTTCAAGGCTTCC-3’ |
|  |  | *Reverse* | 5’-TAAGCCTTGCTTGCTTCGAT-3’ |
| *GAPDH* | | *Forward* | 5’-GCATCCTGGGCTACACTGA-3’ |
|  |  | *Reverse* | 5’-CCACCACCCTGTTGCTGTA-3’ |
